# Unmasking the Deadly Fungus: A Case Report of Rhino-Orbital-Cerebral Mucormycosis in a Diabetic Patient With Unusual Presentation

**DOI:** 10.7759/cureus.36191

**Published:** 2023-03-15

**Authors:** Anas M Aljoaid, Mohammed Khayat, Nashwa Alkhotani

**Affiliations:** 1 Internal Medicine, Al-Noor Specialist Hospital, Makkah, SAU; 2 Infectious Disease, Al-Noor Specialist Hospital, Makkah, SAU

**Keywords:** rhino orbital cerebral, zygomycosis, diabetes type ii, mucor, rhinocerebral mucormycosis

## Abstract

Mucormycosis (zygomycosis) is a rare, rapidly progressive fungal infection that is opportunistic and usually affects immunocompromised individuals, most commonly patients with diabetes mellitus. It is a fatal infection that requires high clinical suspicion and early disease identification. The global burden of mucormycosis is unknown as it is a rare disease. However, the burden of mucormycosis is increasing worldwide, with the emergence of new risk factors and causative agents. In the Saudi population, the discovered cases and the overall prevalence were low. Herein, we present a case of mucormycosis infection aiming to illustrate the clinical characteristics and the management strategy, besides adding another case to the literature.

## Introduction

Mucormycosis (zygomycosis) is a rare and rapidly progressive fungal infection that is opportunistic and usually affects immunocompromised individuals, most commonly those with diabetes mellitus (DM) [1]. It is a fatal infection that requires high clinical suspicion and early identification of the disease, as well as aggressive medical and surgical interventions. The genus most commonly involved in human infections is the *Rhizopus *species [2]. Mucormycosis is characterized by infarction and necrosis of tissues caused by the invasion of the vasculature by hyphae [3]. The infection usually manifests in rhino-cerebral, pulmonary, gastrointestinal, cutaneous, or disseminated forms [4]. The burden of mucormycosis is increasing worldwide, and there has been a transition in the epidemiology of mucormycosis in recent years with the emergence of new risk factors and causative agents. However, in our Saudi population, the discovered cases and the overall prevalence were low [5]. Herein, we present a case of mucormycosis infection aiming to illustrate the clinical characteristics and management strategy and to add the case to the literature.

## Case presentation

A 57-year-old male, known to have uncontrolled type 2 DM and on insulin, presented to the emergency room with complaints of swelling and pain on the left side of his face. The patient had been in his usual state of health until a month ago when he began to experience these symptoms around his left eye, which had worsened over time with no relieving factors. He was unable to open his left eye and had also been experiencing nasal congestion, purulent nasal discharge, and a headache lasting the same duration. There was no history of fever, weight loss, or loss of appetite. He denied any recent infections, hospitalizations, drug or food allergies, or surgical procedures.

On physical examination, the patient appeared unwell with an average body build. He was conscious and oriented to time, place, and person. His blood pressure was 128/77 mmHg, pulse was 88 bpm, respiratory rate was 18/min, and temperature was 37°C. The left side of his face was swollen with periorbital edema, but there was no discharge from the left eye. He had complete left-eye ophthalmoplegia with restricted eye movement, ptosis, and proptosis. Drooping at the angle of the mouth was noted, and the patient was drooling saliva. An ulcer with brown and blackish discoloration was observed on his hard palate, which was likely due to poor dental hygiene.

**Figure 1:**
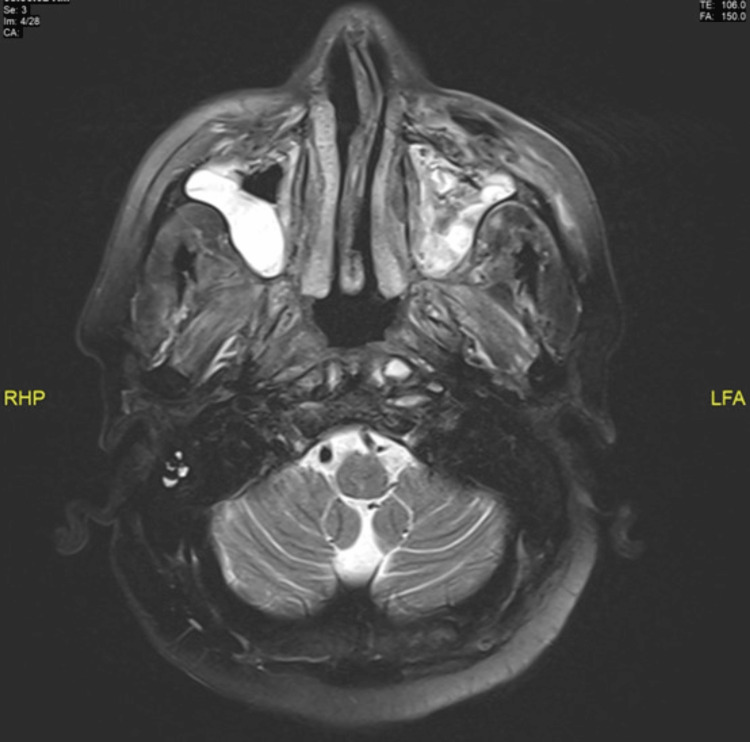
The magnetic resonance imaging (MRI) of the orbits and paranasal sinuses (PNS) showing marked mucosal thickening of the maxillary sinus.

The complete blood count (CBC) with differential showed a white blood cell count (WBC) of 16.0 x 10^9^/L with an absolute neutrophil count of 10.4, a platelet count of 399.00 x 10^9^/L, and a hemoglobin level of 96.5 g/L. The blood glucose level was elevated at 260 mg/dL with no signs of ketoacidosis. The HIV screening test was negative. The erythrocyte sedimentation rate (ESR) was 33 mm/h, and the C-reactive protein (CRP) level was 6.51 mg/L. The HbA1c level was 8.9%, and the Brucella screening test was negative. The magnetic resonance imaging (MRI) of the orbits and paranasal sinuses (PNS) revealed diffuse edema and enhancement involving the face, particularly the orbicularis oris muscles and cheeks, as well as left subcutaneous edema with air foci. Mucosal thickening of the maxillary sinuses was also observed (Figure 1).

**Figure 2:**
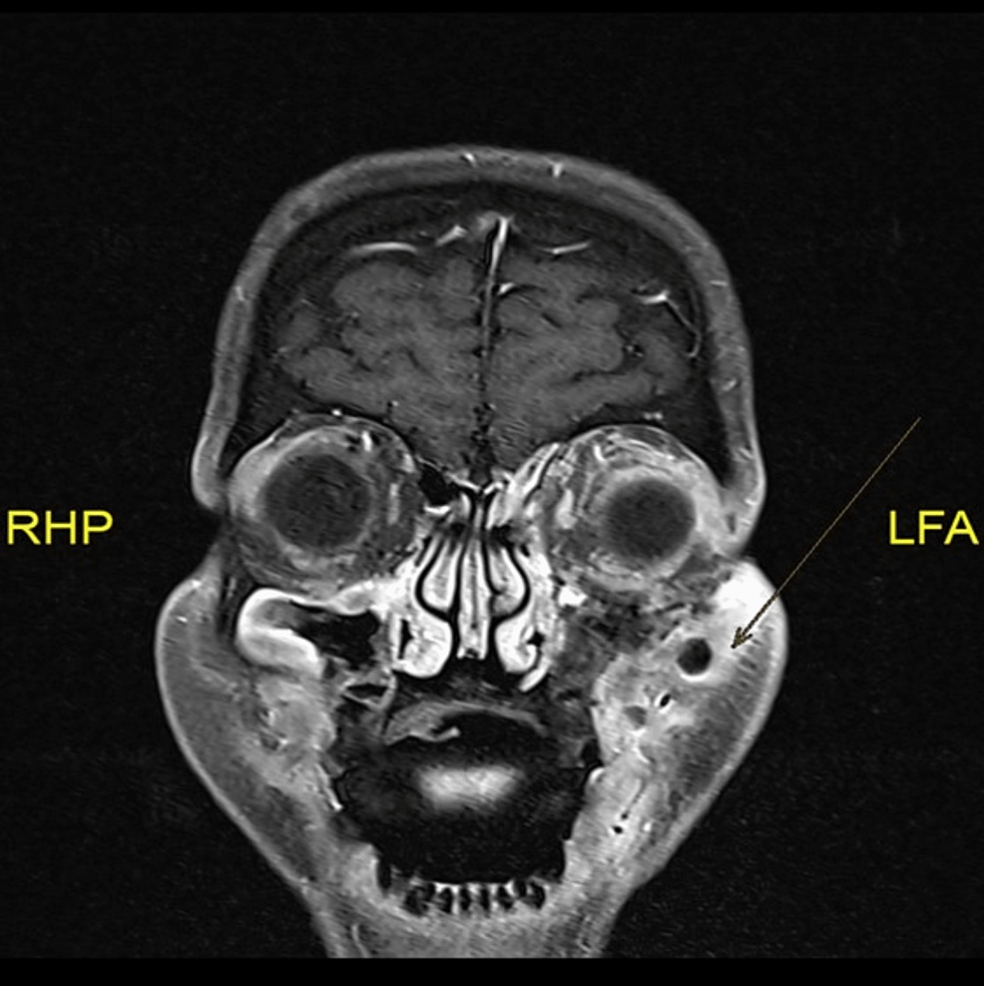
Left cheek collection measuring about 1.5 cm x 1 cm associated with mild diffusion restriction and subcutaneous edema with air foci.

There were diffuse processes of edema and enhancement involving the bilateral retro maxillary areas, bilateral masticator spaces involving the masseter muscle, with a small collection measuring 1.5 cm in the masticator space, bilateral retropharyngeal space with two small collections associated with narrowing of the airway, which, however, remained patent (Figure 2).

**Figure 3:**
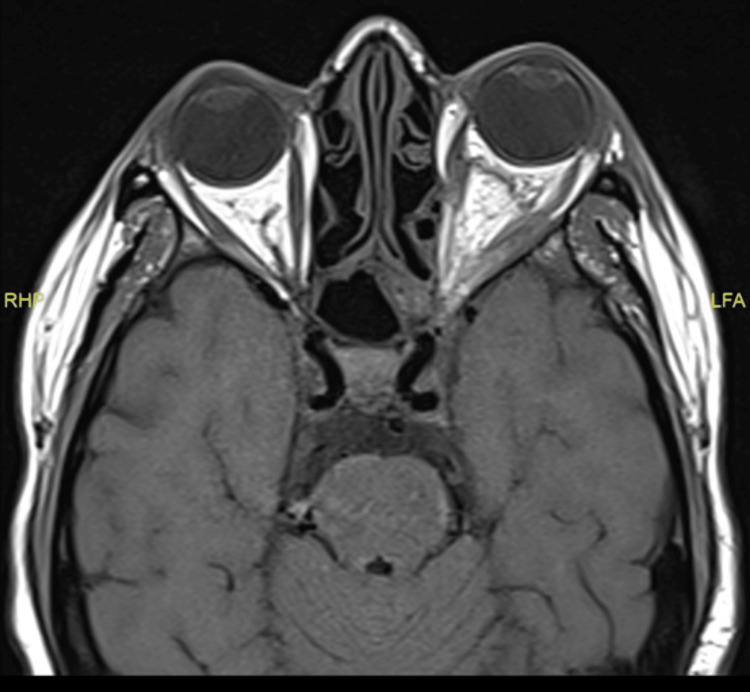
Asymmetrical proptosis of the left eye globe.

The intracranial findings revealed left middle cranial fossa pachymeningeal enhancement with adjacent edema and enhancement of the left Meckel’s cave, mild enhancement of the cisternal left trigeminal nerve, and the skull base bilaterally. In the orbit, the left eye globe exhibited asymmetrical proptosis. The left orbit demonstrated a diffuse process of edema and enhancement, and mild diffusion restriction was seen involving pre- and post-septal fat, left ocular muscles, mainly the inferior rectus, and enhancement of the intraocular part of the optic nerve (Figure 3).

Following the imaging, the patient was transferred to the operating room. The ENT and Maxillofacial surgeons utilized endoscopy to conduct a thorough examination of the bilateral nasal cavities. They noted pus discharge in the inferior and middle meatus of the left side, and necrotic tissue was present on both sides. Biopsies were taken from multiple areas, including normal and necrotic sections of the mucosa covering the septum, as well as from the maxillary crest for histopathological evaluation. All necrotic tissue was then bilaterally debrided, and nasal packing was applied. Subsequently, functional endoscopic sinus surgery (FESS) was performed, which included medialization of the middle turbinate and uncinectomy on the left side, middle meatal antrostomy with the cleaning of the left maxillary sinus, and left anterior and posterior ethmoidectomy, as well as widening of the left frontonasal recess. An incisional biopsy was also taken from the necrotic mucosa of the hard palate.

**Figure 4:**
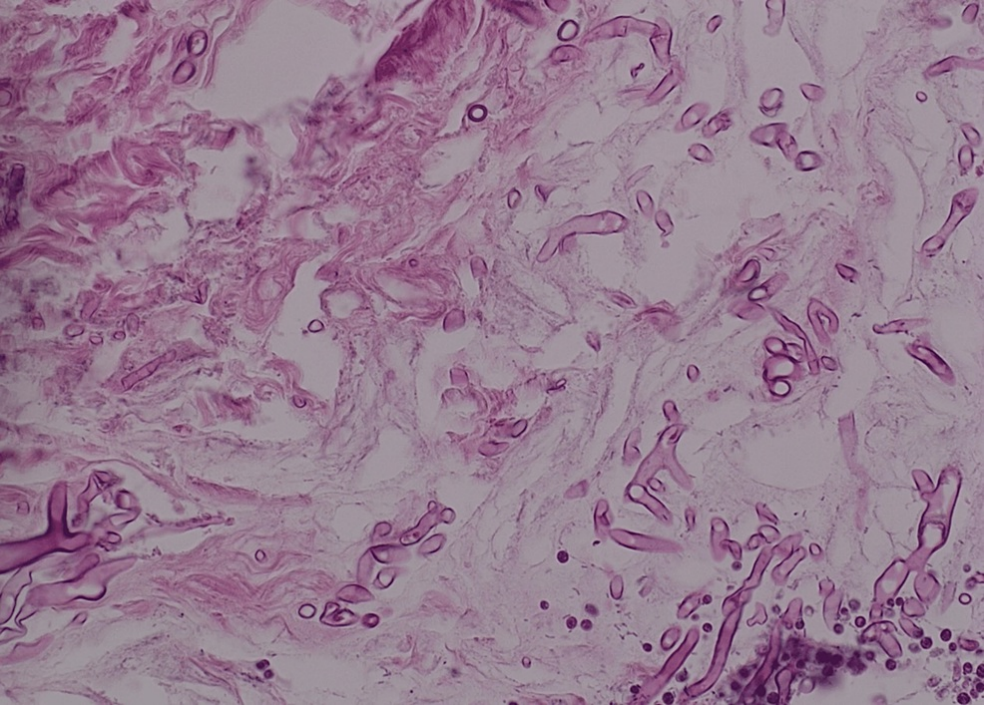
Showing the organism as broad pauciseptate ribbon-like hyphae with an acute angle.

The histopathology report of the sinus and palate biopsies revealed broad pauciseptate ribbon-like hyphae with acute angle branching, which led to a diagnosis of rhino-orbital-cerebral mucormycosis (ROCM) (Figure 4). The patient received treatment with intravenous liposomal amphotericin B at a dose of 5mg/kg/day, and regular debridement led to initial improvement. However, the patient was lost to follow-up.

## Discussion

Mucormycosis is a fungal infection that typically affects immunocompromised patients, such as those with diabetes or receiving chemotherapy. However, it can also occur in immunocompetent individuals but rarely. The disease is caused when the organism penetrates the mucous membrane and has a rapid progression pattern [1,6]. There are five types of mucormycosis, with ROCM being the most common, followed by cutaneous, pulmonary, disseminated, and gastrointestinal mucormycosis, respectively [6,7].

DM is the most common predisposing factor for ROCM, as it alters the normal body response to pathogens, and the hyperglycemic state encourages fungal proliferation [7-9]. Therefore, our patient's diabetic status is consistent with the literature. Another significant risk factor for ROCM is solid organ transplantation [7].

The clinical features of ROCM can vary and include pain and numbness in the face, eye proptosis, vision loss, conjunctival suffusion, soft tissue swelling, or palatal ulcers with necrosis [6,10]. However, our patient presented with facial swelling and pain, periorbital swelling, nasal congestion, purulent nasal discharge, headache, restricted eye movement, ptosis, and proptosis. Additionally, there was drooping at the angle of the mouth with drooling of saliva and an ulcer with necrosis in the hard palate.

Diagnosis of ROCM can be supported with radiographic studies, which may demonstrate opacification and bony infiltration of the sinuses [8]. A computed tomography (CT) scan should be performed immediately to evaluate the PNS, and MRI should be used if orbit or brain involvement is suspected [6,9]. The definitive diagnosis is established through histopathology, which involves biopsy from the involved area, and it will show colonies of broad pauciseptate ribbon-like hyphae with an acute angle [9]. This was the same finding in our patient.

The management of ROCM should be a combination of surgical and medical treatment, primarily involving surgical debridement and administration of amphotericin B [8,9]. This was the primary approach used in the present case, and the patient showed improvement at that time. Further management and optimization of blood sugar levels should be established, and the patient should be followed up to prevent or manage recurrence if it occurs.

## Conclusions

Controlling diabetes can improve immunity and, as a result, reduce the incidence of opportunistic infections such as mucormycosis. Early diagnosis and aggressive management, which consist of surgical debridement and antifungal treatment, can increase the survival rate in ROCM.
